# From symptom onset to ED departure: understanding the acute care chain for patients with undifferentiated complaints: a prospective observational study

**DOI:** 10.1186/s12245-024-00629-x

**Published:** 2024-04-15

**Authors:** Lieke Claassen, Laura Magdalena Ritter, Gideon Hubertus Petrus Latten, Noortje Zelis, Jochen Willo Lennert Cals, Patricia Maria Stassen

**Affiliations:** 1 Department of Emergency Medicine, Zuyderland Medical Centre, Heerlen, The Netherlands; 2 Department of Internal Medicine, Division General Medicine, Section Acute Medicine, Maastricht University, Maastricht, The Netherlands; 3 Department of Family Medicine, Care and Public Health Research Institute (CAPHRI), Maastricht University, Maastricht, The Netherlands; 4 Department of Internal Medicine, Division General Medicine, Section Acute Medicine, Cardiovascular Research Institute Maastricht (CARIM), Maastricht University, Maastricht, The Netherlands

**Keywords:** Acute care chain, Patient journey, Undifferentiated complaints

## Abstract

**Background:**

For most acute conditions, the phase prior to emergency department (ED) arrival is largely unexplored. However, this prehospital phase has proven an important part of the acute care chain (ACC) for specific time-sensitive conditions, such as stroke and myocardial infarction. For patients with undifferentiated complaints, exploration of the prehospital phase of the ACC may also offer a window of opportunity for improvement of care. This study aims to explore the ACC of ED patients with undifferentiated complaints, with specific emphasis on time in ACC and patient experience.

**Methods:**

This Dutch prospective observational study, included all adult (≥ 18 years) ED patients with undifferentiated complaints over a 4-week period. We investigated the patients’ journey through the ACC, focusing on time in ACC and patient experience. Additionally, a multivariable linear regression analysis was employed to identify factors independently associated with time in ACC.

**Results:**

Among the 286 ED patients with undifferentiated complaints, the median symptom duration prior to ED visit was 6 days (IQR 2–10), during which 58.6% of patients had contact with a healthcare provider before referral. General Practitioners (GPs) referred 80.4% of the patients, with the predominant patient journey (51.7%) involving GP referral followed by self-transportation to the ED. The median time in ACC was 5.5 (IQR 4.0-8.4) hours of which 40% was spent before the ED visit. GP referral and referral to pulmonology were associated with a longer time in ACC, while referral during evenings was associated with a shorter time in ACC. Patients scored both quality and duration of the provided care an 8/10.

**Conclusion:**

Dutch ED patients with undifferentiated complaints consulted a healthcare provider in over half of the cases before their ED visit. The median time in ACC is 5.5 h of which 40% is spent in the prehospital phase. Those referred by a GP and to pulmonology had a longer, and those in the evening a shorter time in ACC. The acute care journey starts hours before patients arrive at the ED and 6 days of complaints precede this journey. This timeframe could serve as a window of opportunity to optimise care.

**Supplementary Information:**

The online version contains supplementary material available at 10.1186/s12245-024-00629-x.

## Background

Within the acute care chain (ACC), healthcare providers, such as General Practitioners (GPs), Emergency Medical Services (EMS) and Emergency Department (ED) professionals, all aim to provide optimal and timely care for patients. For specific time-sensitive conditions (e.g. stroke, major trauma and myocardial infarction), these professionals work closely together in carepathways, hereby succesfully reducing any delay in appropriate treatment and improving outcomes [1–4]. These specific time-sensitive conditions, however, are relatively easy to recognise early and comprise only a minority of all patients that visit the ED. In contrast, many other conditions are more difficult to recognise, although these concern a substantial proportion of the ED population. Recognising time-sensitive conditions, such as sepsis, early in the ACC is necessary to improve care for this group of patients as well [5, 6]. 

Recognition of less specific time-sensitive conditions early in the ACC is, however, challenging. There is a lack of studies on this topic in the prehospital phase and studies in the ED are scarce. Besides, the results of studies on this topic likely depend on the selection of ED patients, which reflects differences in healthcare systems worldwide. One study from Denmark, with a comparable acute care system as the Netherlands, showed that of all adult ED patients, only 35% were referred with a presumptive diagnosis [7–9]. A study in Switzerland (where there are more self-referrals than in the Netherlands) found that up to 20% of patients presented with even more undifferentiated complaints (e.g. general weakness), generally referred to as nonspecific complaints. An acute medical problem requiring emergency medical intervention can ultimately be identified in over 50% of these patients [6, 10, 11]. These data show how hard it is to recognise time-sensitive conditions early. However, previous studies on specific time-sensitive conditions demonstrated substantial improvements in both treatment (e.g. early initiation of MONA therapy) and logistic efficiency (e.g. transfer to a catheterization room) [12, 13]. For improvement of care for patients with undifferentiated complaints, similar to care of patients with myocardial infarction, it is important to first explore the entire ACC and to investigate factors associated with the time in ACC [14]. 

We further believe that in order to optimise care, it is also important to evaluate how undifferentiated patients experience their journey and time in the ACC. Previous studies focused solely on the ED, and these indicated that spending time in the ED can be very stressful for patients [15–17]. 

In this prospective exploratory study, we therefore aimed to explore the characteristics and journey through the entire ACC of adult ED patients with undifferentiated complaints. We will specifically focus on time in ACC, on factors associated with this time, and on the patients’ experience regarding their journey.

## Methods

### Design and setting

This prospective observational study took place during a 4-week period between March 15th 2021 and April 11th 2021 in Zuyderland Medical Centre, a large teaching hospital with two EDs in the Netherlands. The combined annual census of these 2 EDs is approximately 45,000. In the Netherlands, the first step in emergency care is usually provided by GPs, either in their practices during office hours or in general practitioner centres during out-of-hours. Access to emergency care in the hospital requires either a referral from the GP or a direct transfer by EMS. Patients are discouraged to visit the ED on their own initiative (for more details: [18, 19]).

### Patients

All adult (≥ 18y) patients who visited 1 of the 2 participating EDs with undifferentiated complaints were eligible for inclusion. Patients were approached for participation when research staff was present. Most patients were approached during the second half of their stay in the ED. Written informed consent had to be obtained prior to inclusion.

We included adult patients with undifferentiated complaints (e.g. dyspnoea, fever, abdominal pain, general weakness). We excluded those who visited the ED with the following (suspected) specific conditions: stroke, myocardial infarction, ruptured aneurysm and major trauma, and excluded patients primarily presenting for the following specialties: vascular surgery, traumatology, cardiology and neurology. Patients who were unable to understand the questionnaire were excluded. Patients visiting the ED more than once during the study period were included at their first presentation only, as a second visit to the ED may have different patterns.

### Data collection

Demographics and time in ACC data were collected from medical records (including hospital files, referral letters and EMS notes) and from a questionnaire filled out by the patient using a Case Report Form (Appendix A, supplementary file 1).

ACC was categorised into three phases of the ACC: the pre-referral phase, the referral phase and the hospital phase. In addition to data on duration of complaints, prior contacts with healthcare providers, prescribed medication prior to ED visit and part of the day of referring contact we defined four different patient journeys:

- GP referral + own transportation [1].
- GP referral + EMS transportation [2].
- Self-referral + own transportation [3].
- Calling 112 (national emergency number) + EMS transportation [4].

**Fig. 1:**
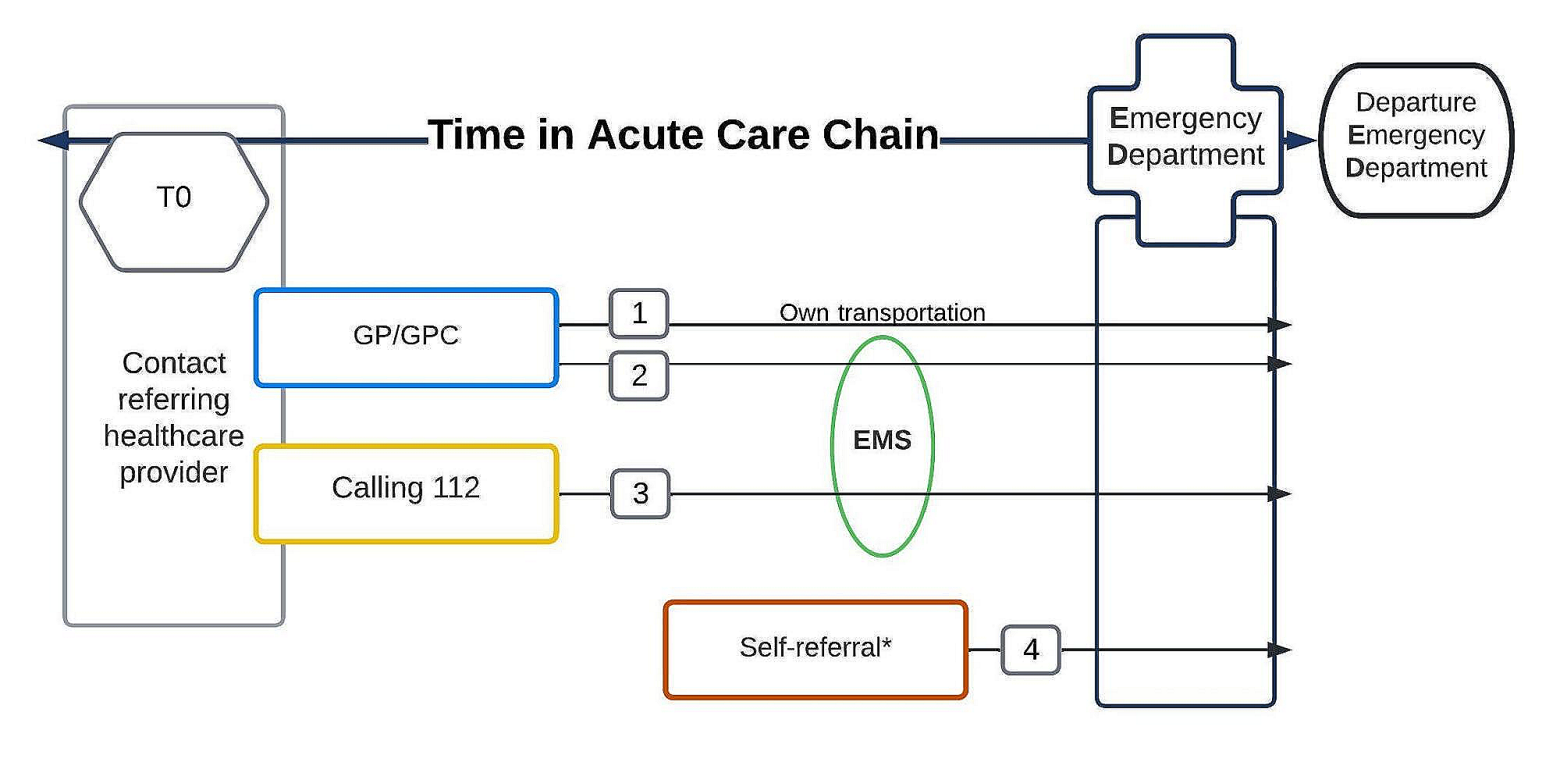
Visualisation of the acute care chain Abbreviations: T0 - start of the ACC; GP - general practitioner, GPC - General Practitioner Cooperative, EMS -emergency medical services *In case of self-referral and no previous contact with a healthcare provider, the time of registration for the ED was considered to be T0

Collected data on the hospital phase were: referred specialty, presenting complaints, ED triage urgency (based on the Manchester Triage System) [20], and ED arrival and departure time. An overview of the ACC is provided in Fig. 1.

The time in ACC was defined as the time between the moment of contacting the referring healthcare provider (physical or telephonic consultation) and the time the patient left the ED. In case of self-referral and no previous contact with a healthcare provider, the time of registration for the ED was considered to be T0.

Patients were also questioned regarding their impression on quality, duration, and timing of the provided care (Appendix A, supplementary file 1).

### Analysis and statistics

We performed descriptive analyses of the variables of the pre-referral, the referral and the hospital phase. Continuous variables were reported as means with standard deviations (SD) or medians with interquartile ranges (IQRs). Categorical variables were reported as absolute numbers and valid percentages were used when values were missing.

Univariable linear regression analysis was used to assess the association between time in ACC and the following variables: demographic variables (age and sex), pre-referral variables (duration of complaints, prescribed medication in this disease episode prior to current ED visit (yes/no)), referral variables (part of the day of referring contact and patient journey (GP referral, EMS transportation)) and hospital variables (referred specialty, complaints and triage urgency). For categorical variables, dummy variables were created. We performed a multivariable linear regression analysis using the forced entry method, including all the variables analysed in the univariable analysis. The unstandardized regression coefficient (B) and 95% confidence interval were calculated.

We used a Pearson correlation test to investigate the association between the reported experience and the time in ACC and triage urgency.

We compared demographic variables (age and sex) and triage urgency of included and non-included patients to investigate for possible selection bias. To compare included with non-included patients we used Students’ T test, Mann Whitney U test, One way Anova or Kruskal Wallis test for continuous data when appropriate. For comparison of categorical data, the Chi-square or Fisher exact test was used. A *p*-value < 0.05 was considered significant. All statistical analyses were performed using IBM SPSS statistical software version 26 (Chicago, Illinois, USA).

We aimed to include a minimum of 200 patients. No sample size/power calculation was performed for this exploratory study. We used the Strengthening the Reporting of Observational Studies in Epidemiology guidelines for reporting this observational study [45]. The study was approved by the medical ethics committee of Zuyderland (METC-Z nr. 20,200,198).

## Results

### Participants

**Fig. 2:**
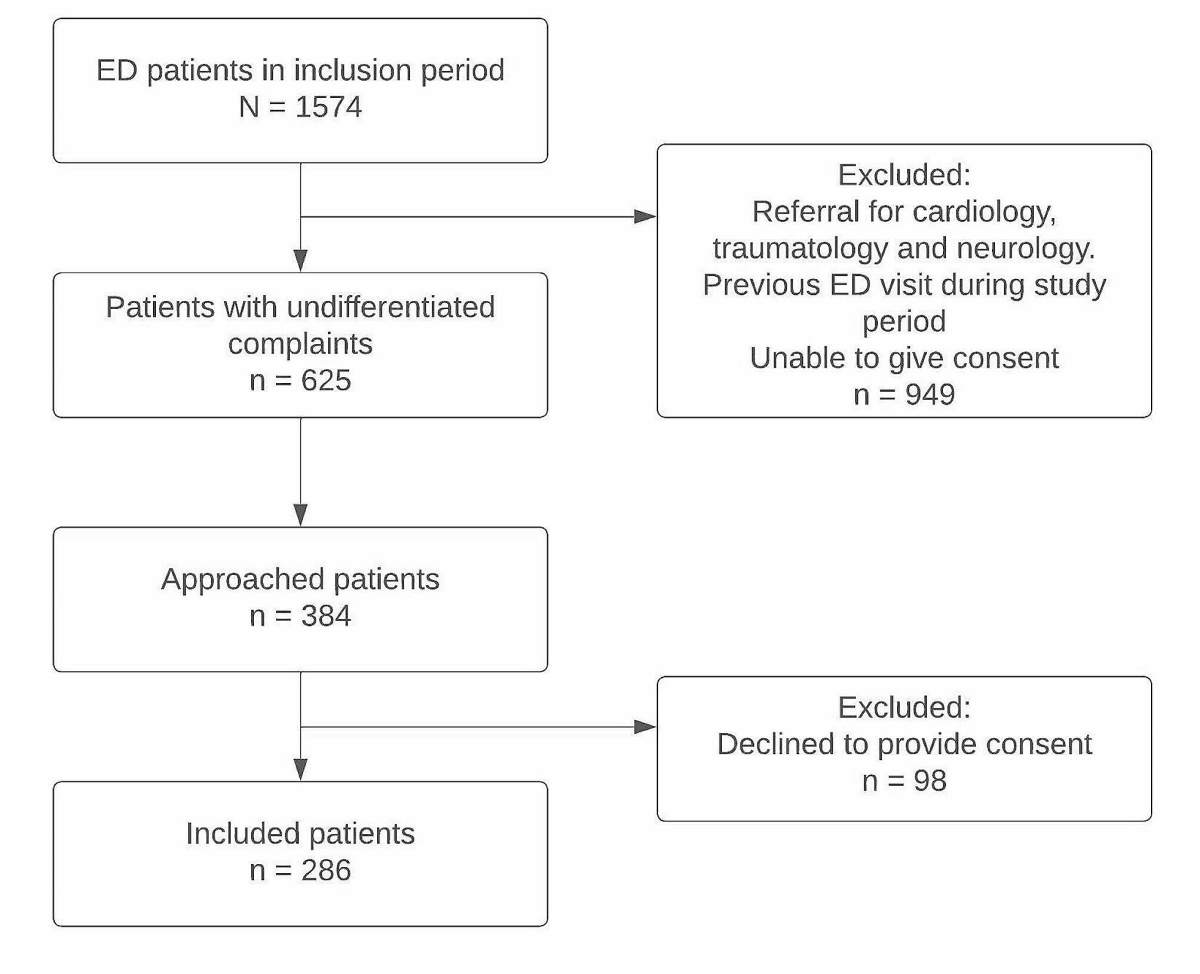
Flowchart of study population Abbreviations: ED – Emergency Department

During the inclusion period, 625 eligible patients presented to the ED, of which 384 (61.4%) were asked to participate (Fig. 2). Of these, 286 (45.8%) patients were included.

### Baseline characteristics

The median age of the included patients was 65 (IQR 52–75) years (Table 1). Patients had complaints for a median of 6 (IQR 2–10) days before ED presentation. In this phase, 58.6% of patients had contacted a healthcare provider at least once, while 32.3% had been prescribed medication.

**Table 1:** Baseline characteristics

*N* = 286	Median (IQR) or n (%)
**Demographics**	
**Age**	65 (IQR 52–75)
**Female**	163 (43.0%)
**Pre-referral phase**	
**Duration of complaints (days)**	6 (IQR 2–10)
≤ 1 day	36 (12.6%)
1–30 days	222 (77.6%)
> 30 days	28 (9.8%)
**1 or more contact with healthcare provider**	166 (58.0%)
Number of contacts (*n* = 164^a^)	2 (IQR 1–3)
**Prescribed medication**	92 (32.2%)
**Referral phase**	
**Part of the day of referring contact**	
Day (8–17 h)	255 (89.2%)
Evening (17–23 h)	22 (7.7%)
Night (23–8 h)	9 (3.1%)
**GP referral**	230 (80.4%)
**EMS transportation**	96 (33.6%)
**Patient journey**	
GP referral + own transportation	148 (51.7%)
GP referral + EMS transportation	82 (28.7%)
Self-referral + own transportation	42 (14.7%)
Calling 112 + EMS transportation	14 (4.9%)
**Hospital phase**	
**Referred specialty**	
Pulmonology	101 (35.3%)
Internal medicine* + Gastroenterology	99 (34.6%)
Surgery + orthopaedics**	56 (19.6%)
Other	30 (10.5%)
**Presenting complaints**	
Dyspnoea	109 (38.1%)
Abdominal pain	96 (33.6%)
General malaise	32 (11.2%)
Other	49 (17.1%)
**Diagnosed with COVID-19**	47 (17.1%)
ED triage urgency (*n* = 278^b^)	
Red	1 (0.4%)
Orange	72 (25.9%)
Yellow	133 (47.8%)
Green	72 (25.9%)
Blue	0
Highly urgent (red/orange)	73 (26.3%)
Highly urgent + EMS transportation	38 (52.1%)
Urgent (yellow/green/blue)	205 (73.7%)
Urgent + EMS transportation	57 (27.8%)

*Values* are n (%) for ordinal variables and median (IQR) for continues variables

*Abbreviations*: IQR – interquartile range, GP – general practitioner, EMS – emergency medical services, ED – emergency department, MTS – Manchester Triage System ^a^ Two missing. ^b^ Eight no triage.

*Definitions*: ED triage urgency – triage categories according to MTS were defined as highly urgent (red and orange) and urgent (yellow, green and blue).

* Internal medicine including geriatrics

** Non-trauma

**Fig. 3:**
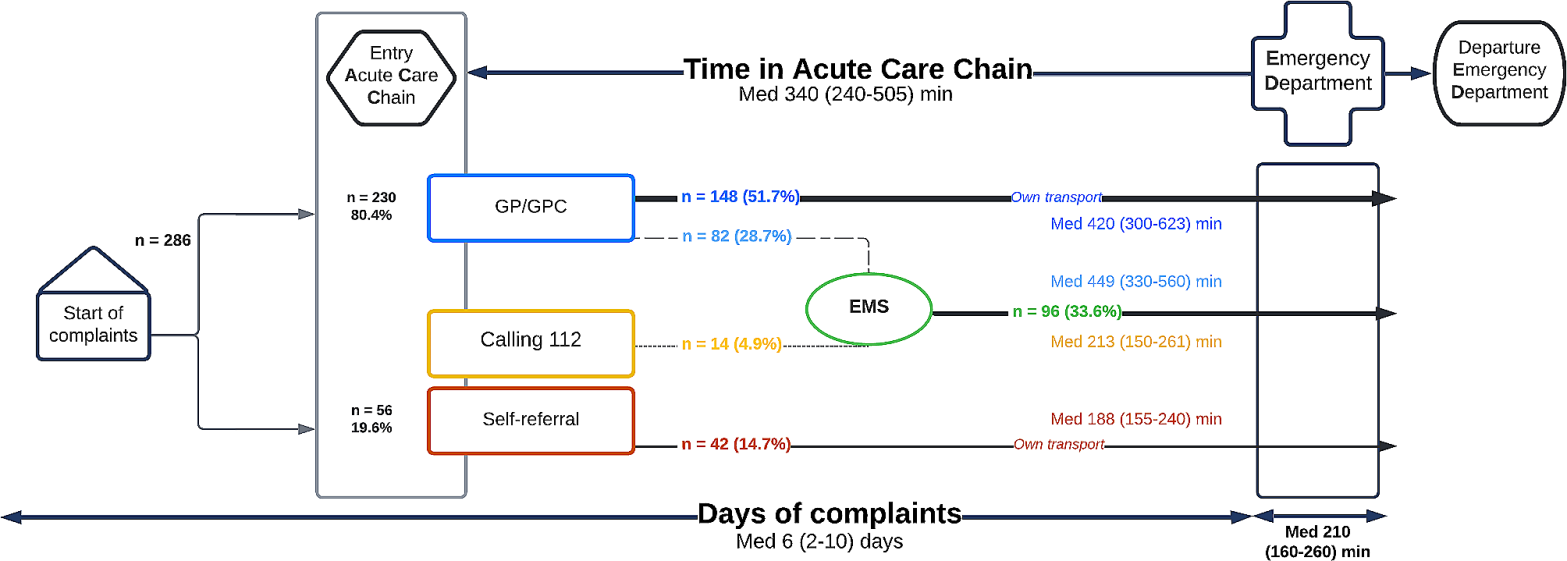
Patient journey of adult ED patients with undifferentiated complaints and the time spent in ACC. Abbreviations: GP – general practitioner, GPC – General Practitioner Cooperative, EMS – emergency medical services, med – median, IQR – interquartile range, min – minutes

GPs referred 80.4% of all patients, and 33.6% were transported by EMS. Overall, the predominant patient journey was GP referral + own transportation (51.7%, Fig. 3).

In the ED, 25.0% of patients were triaged as highly urgent, with dyspnoea as most common (38.1%) presenting complaint. Most highly urgent patients (52.1%) were transported by EMS, while the minority of patients with lower urgency (27.8%) were.

### Time in ACC

The median time in ACC was 340 (IQR 240–505) minutes, approximately 5.5 h. Of these, 210 (IQR 160–260) minutes (3.5 h) were spent in the ED (Fig. 3). The longest time in ACC was the journey GP referral + EMS transportation, where patients spent 449 (IQR 330–580) minutes. The shortest journey was self-referral with 188 (IQR 155–240) minutes.

### Associations between demographic, prehospital and hospital factors and time in ACC

Table 2 shows the results of the linear regression analysis regarding the association of different factors with the time in ACC. All assumptions for linear regression were checked and no violations were detected.

**Table 2:** Factors associated with time in ACC in adult ED patients with undifferentiated complaints

Variable	Univariable			Multivariable		
	B	95% CI	*P*-value	B	95% CI	*P*-value
Constant				206		0.013
Age	1	0–2	0.236	1	45–367	0.459
Female sex	10	-32–52	0.648	2	-46–50	0.929
Complaints (days)	0	-3–2	0.806	-1	-4–2	0.445
Prescribed medication*	-12	-66–41	0.646	-1	-50–48	0.970
Daypart contact**						
Evening	-157	-233 – -81	< 0.001	-109	-194 – -23	0.013
Night	-50	-169– 69	0.410	32	-123–186	0.686
GP Referral	223	177–268	< 0.001	199	135–263	< 0.001
EMS transportation	21	-23–65	0.358	-15	-71–42	0.615
Referred specialty						
Pulmonology	101	59–143	< 0.001	73	5–140	0.035
Surgery + orthopaedics	-72	-124 – -20	0.006	-29	-102–43	0.422
Other	-78	-145 – -11	0.023	-40	-137–58	0.422
Complaints						
Dyspnoea	104	63–145	< 0.001	-3	-81–75	0.937
General malaise	-5	-71–61	0.878	-38	-124–47	0.379
Other	-118	-172 – -65	< 0.001	-20	-103–62	0.627
Urgency: urgent	-42	-2–6	0.087	-23	-81–34	0.424

R^2^ = 0.347

Abbreviations: ACC – acute care chain, ED – emergency department, *B* – unstandardized regression coefficient, CI – confidence interval, GP – general practitioner, EMS – emergency medical services; *R*^*2*^ – coefficient of determination

* Prescribed medication in this disease episode prior to current ED visit. ** Part of the day of referring contact

In the univariable analysis, contacting the referring healthcare provider in the evening, referral to surgery/orthopaedics, and the group of “other” presenting complaints, were associated with a shorter duration in ACC. GP referral, referral to pulmonology and dyspnoea were associated with a longer duration in ACC.

The multivariable regression analysis showed that first contact with the referring healthcare provider during the evening was independently associated with a shorter time in ACC (Table 2). GP referral and referral to pulmonology were associated with a longer time in ACC.

During the evening, the ACC was shorter than during the day/night, mainly because of a significantly shorter prehospital phase in the evening compared to during the day/night (32 vs. 130 min, *p* < 0.001), while the ED-phase was shorter, but not significantly different (180 vs. 210 min, *p* = 0.201).

The overall model was significant (F(15, 149) = 5.290; *p* < 0.001) and accounted for 34.7% of the variance of the time in ACC.

### Patient experience

The median score on the quality of the care process was 8 out of 10 (IQR 8–9). The median score for the duration of the journey was also 8 out of 10 (IQR 8–9). Overall, 76.6% (*n* = 219) of patients judged the timing of referral to be correct, 18.9% (*n* = 54) judged they were referred too late, 3.1% (*n* = 9) judged that they were referred too early and 1.4% (*n* = 4) did not answer this question.

No associations were found between perceived quality and duration and the time in ACC (p *=* 0.770 and *p* = 0.350) nor with triage urgency (*p* = 0.379 and *p* = 0.544).

### Comparison of included with non-included patients

We found no significant differences in age and ED triage urgency between the included patients and eligible, but non-included patients (*n* = 339). Included patients, however, were more often female (57.0% vs. 44.4%, *p =* 0.002) than non-included patients (Supplementary Table 1, Additional file 2).

## Discussion

In this prospective study, we investigated adult ED patients with undifferentiated complaints, with a focus on their time in and their journey through the ACC. We found that on the day of ED visit, patients spent more than 5.5 h in the ACC, with 40% of that time spent in the prehospital phase. Patients had complaints for a median of 6 days prior to the ED visit, during which almost 60% already had contact with a healthcare provider before their referral contact and one third had been prescribed medication before their ED visit. In addition, over 80% of patients were referred to the ED by a GP and one third was transported by EMS. This indicates that the prehospital phase comprises a substantial part of the ACC for patients with undifferentiated complaints in terms of time and contacts with healthcare providers.

To the best of our knowledge, there are no studies on the time spent in the ACC in patients with undifferentiated complaints. In this study we showed that prior to ED arrival, patients spent about two hours, a substantial amount of time (40%), in the prehospital setting, and the median time of symptoms before arrival at the ED was even 6 days. In this phase, in many patients (32.2%) medication was prescribed. The phase before the patient enters the ED may offer opportunities for optimising care. An example is that risk stratification can be optimised, and tools to help healthcare providers can be developed [21, 22]. A Swedish study on the quality of prehospital decision-making for referral to alternative levels of care by EMS showed that only 1% of patients not transported by EMS were diagnosed with a time-sensitive condition, which shows that prehospital professionals contribute to optimising care efficiency, but may be in need for extra tools to help them [23]. other options might be to identify those at risk for an ED visit and increase home care, initiate treatment (e.g. nebulisation of bronchodilators, injection of diuretics or individualised antibiotic treatment) or stimulate self-management strategies in these patients. The provided insight in the prehospital phase – duration and involvement of professionals – shows that it is worthwhile to look for opportunities in this phase for optimising care.

Since there are no studies on time in ACC we can only compare the ED-LOS with other studies. We found a longer ED-LOS (50 min) than compared to other Dutch studies [24–26]. Which is probably due to differences in patient selection (our patients were relatively old, presented with undifferentiated complaints and often COVID-19 (17.1%)) [27–29]. Remarkably, the ED-LOS outside the Netherlands is almost as long as or longer than the total time in ACC in our study, which may be indicative of the efficiency of our acute care organisation [30–32]. 

Three factors were independently associated with time in ACC: [1] contact with a healthcare provider during the evening leading to a shorter time in ACC [2], referral by GP and [3] referral to pulmonology, both leading to a longer time in ACC. An explanation for the shorter time in ACC in the evening, mainly in the prehospital phase, could be that [1] in the evening more self-referrals present to the ED (post-hoc analysis on self-referrals: 27.3% during evenings vs. 13.6% outside evenings) and [2] during out-of-hours, the GP on call is stationed next to the ED of the hospital, which minimises the transport time to the ED. Spending less time in the ED when being referred in the evening is in contrast with other studies [29, 33]. 

A longer journey in the ACC after referral by a GP is logical compared to those who self-refer. Interestingly we found a relatively high percentage of patients referred by a GP in our study compared to other Dutch studies (80.4% vs. 56–76%) [25, 34, 35]. We hypothesise that this difference is caused by exclusion of patients with differentiated complaints, such as stroke or trauma, as these complaints more often warrant self-referral or an 112 call [24]. The longer time in ACC for pulmonology patients is likely due to the higher than average need for diagnostic imaging and the high incidence of COVID-19 in our cohort (17.1%) [36, 37]. 

The combined variables (age, sex, duration of complaints, prescribed medication, daypart, GP referral, EMS transportation, referred specialty, presenting complaints and ED triage urgency) collectively could explain one third of the time in ACC. This is in line with studies on ED-LOS reporting that with many factors, such as logistical factors (e.g. bed shortage, waiting time for radiology) and factors beyond the influence of the ED are of influence [33, 38, 39]. 

Our patients, perceived both quality and duration of the provided care as good (8/10). Interestingly, no association was found between perceived quality of care and time in ACC, which is not in line with other studies where prolonged waiting times in the ED were associated with worse experience [40, 41]. Also, no association was found with urgency levels, in contrast with another study, which showed lower patient satisfaction in patients triaged as non-urgent [42]. A probable explanation for the high perceived quality may be that time in ACC was not the only factor of influence. For example, quality of nursing care is a strong predictor of patients experience, as is relief of symptoms [43, 44]. A qualitative study is needed to investigate this issue in more depth.

### Strengths and limitations

A strength of our study is that, to the best of our knowledge, we are the first to investigate the entire ACC of patients with undifferentiated complaints, which comprise a significant proportion of the ED population. A possible limitation could be the use of a convenience sample, resulting in a relatively small group of patients presenting out-of-hours. To investigate whether this caused selection bias, we compared the included with the non-included patients and found no differences in age and triage urgency, only in sex. Nevertheless, it is possible that some associations were not accounted for in our analysis.

In order to optimise inclusions in future studies, investigators could consider to extend the informed consent period to 72 h after presentation and 24/7 availability of a research team. One should realise, however, that all prospective studies on this topic will require an enormous effort.

## Conclusion

Patients with undifferentiated complaints presenting to the ED had a time in ACC of 5.5 h, with almost 40% of that time spent in the prehospital phase. During their 6 days of complaints before ED visit, more than half had prior contact with a healthcare provider and the most common patient journey was GP referral and self-transportation to the ED. Referral by GP and referral to pulmonology were associated with a longer time in ACC, and referral in the evening with a shorter time in ACC. Patients were generally satisfied with the quality and duration of care, independent of their time in ACC.

The acute care journey starts hours before patients arrive in the ED. Future research should further investigate the phase before patients eventually end up in the ED because it could serve as an important window of opportunity to optimise care.

### Electronic supplementary material

Below is the link to the electronic supplementary material.

Supplementary Material 1

Supplementary Material 2

## Data Availability

The database generated and analysed during the current study are not publicly available due to participant anonymity issues but are available from the corresponding author on reasonable request from.

## Abbreviations

ACC: Acute Care Chain
CI: Confidence Interval
CT: Computed Tomography
ED: Emergency Department
ED-LOS: Emergency Department Length Of Stay
EMS: Emergency Medical Services
GP: General Practitioner
GPC: General Practitioner Cooperative
IQR: Interquartile Range
Med: Median
METC: Medical Ethics Committee
MTS: Manchester Triage System
SD: Standard Deviations
TACC: Time in the Acute Care Chain
