# Supplementary material for: From symptom onset to ED departure: understanding the acute care chain for patients with undifferentiated complaints: a prospective observational study
Source: Int J Emerg Med. 2024 Apr 15;17:55. doi: 10.1186/s12245-024-00629-x (PMC11020825; doi:10.1186/s12245-024-00629-x)
Supplement: Supplementary file 1 — Supplementary Material 1 [file 12245_2024_629_MOESM1_ESM.docx]

**Additional file 1**

English version

| **Questionnaire TACC study – T**ime within the **A**cute **C**are **C**hain |
| --- |

Dear sir/madam,

We would like to know more about what happened from the moment you became ill until your emergency department visit with us right now. We would like to do this through a few questions, which will only take a few minutes of your time. We thank you in advance for participating in this survey.

| GENERAL INFORMATION | | |
| --- | --- | --- |
|  | Sex | ⃝ Male  ⃝ Female |
|  | Age | ………… years |
|  | Date (at the time of entry into the ED) | ……-……-………… |

| YOUR VISIT TO THE EMERGENCY DEPARTMENT (ED) TODAY | | |  |
| --- | --- | --- | --- |
|  | With what complaint do you visit today? | …………. |  |
|  | Referred specialty: | …………. |  |
|  | Were you referred by a general practitioner (GP) today? | ⃝ Yes; go to question 7  ⃝ No, go to question 9 |  |
|  | What time did you contact the GP today? | Time: ……:…… h |  |
|  | What time did you visit the GP today, or did they come to your home? | Time: ……:…… h |  |
|  | Did you come to the emergency room by ambulance today? | ⃝ Yes; go to question 10.  ⃝ No: go to question 11. |  |
|  | What time was the ambulance at your place today? | Time: ……:……h |  |
|  | Time of ED arrival: | Time: ……:…… h |  |
|  | Time of ED departure: | Time: ……:…… h |  |
|  | What was the final diagnosis made by the doctor in the ED? | ………………………………………………………………………………. |  |

| **YOUR EXPERIENCE REGARDING THE TRAJECTORY TODAY** | |
| --- | --- |
|  | How satisfied are you with the duration of the process so far? That is, from the time you contacted a health care provider today to treatment in the emergency room?   \| Very unsatisfied \| 1 \| 2 \| 3 \| 4 \| 5 \| 6 \| 7 \| 8 \| 9 \| 10 \| Very satisfied \| \| --- \| --- \| --- \| --- \| --- \| --- \| --- \| --- \| --- \| --- \| --- \| --- \| \|  \| ⃝ \| ⃝ \| ⃝ \| ⃝ \| ⃝ \| ⃝ \| ⃝ \| ⃝ \| ⃝ \| ⃝ \|  \| |
|  | How satisfied are you with the quality provided so far? That is, from the time you contacted a health care provider today to treatment in the emergency room?   \| Very unsatisfied \| 1 \| 2 \| 3 \| 4 \| 5 \| 6 \| 7 \| 8 \| 9 \| 10 \| Very satisfied \| \| --- \| --- \| --- \| --- \| --- \| --- \| --- \| --- \| --- \| --- \| --- \| --- \| \|  \| ⃝ \| ⃝ \| ⃝ \| ⃝ \| ⃝ \| ⃝ \| ⃝ \| ⃝ \| ⃝ \| ⃝ \|  \| |
|  | Do you see room for improvement in today's journey? So from the time you contacted a health care provider today until treatment in the emergency room?  ………………………………………………………………………………………………………………………………………………………………………………………  ………………………………………………………………………………………………………………………………………………………………………………………  ………………………………………………………………………………………………………………………………………………………………………………………  ………………………………………………………………………………………………………………………………………………………………………………………  ……………………………………………………………………………………………………………………………………………………………………………………… |

| BEFORE YOUR VISIT AT THE EMERGENCY DEPARTMENT TODAY | | | | |
| --- | --- | --- | --- | --- |
|  | How many days in total did you have complaints before coming in today? | | | ⃝ Complaints since today  ⃝ ………… days of complaints  ⃝ more than 1 month of complaints |
|  | Have you been in contact with a GP, ambulance or physician in a hospital since onset of complaints? | | | ⃝ Yes; go to question 19  ⃝ No; go to question 21 |
|  | Since then, how often have you been in contact with a GP, ambulance, or physician at the hospital? | | | ……………… time(s) |
|  | Were you already prescribed medication? | ⃝ No  ⃝ Yes, namely: …………………………………………… | | Date: ……-……-…………  Time: ……:…… h |
|  | Do you feel you came/were referred to the ED at the right time? | | ⃝ Yes  ⃝ No, I should have been here earlier  ⃝ No, I didn't need to be here right now | |

| **GENERAL** | |
| --- | --- |
|  | Did you miss a question that could have told us something about your experience so far? If so, what question?  ………………………………………………………………………………………………………………………………………………………………………………………  ………………………………………………………………………………………………………………………………………………………………………………………  ………………………………………………………………………………………………………………………………………………………………………………………  ………………………………………………………………………………………………………………………………………………………………………………………  ……………………………………………………………………………………………………………………………………………………………………………………… |

Dutch Version

| **Vragenlijst TACC studie – T**ime within the **A**cute **C**are **C**hain |
| --- |

Beste dame/heer,

Graag willen we meer weten over wat er gebeurd is vanaf het moment dat u ziek werd totdat u nu bij ons op de spoedeisende hulp (SEH) gekomen bent. Dit willen we graag doen door middel van een aantal vragen, die u slechts enkele minuten van uw tijd zullen kosten. Wij danken u bij voorbaat voor deelname aan dit onderzoek.

| ALGEMEEN | | |
| --- | --- | --- |
|  | Geslacht | ⃝ Man  ⃝ Vrouw |
|  | Leeftijd | ………… jaar |
|  | Datum (ten tijde van binnenkomst op SEH) | ……-……-………… |

| UW BEZOEK AAN DE SPOEDEISENDE HULP (SEH) VANDAAG | | | |
| --- | --- | --- | --- |
|  | Met welke klacht komt u vandaag? |  | |
|  | Verwezen specialisme: |  | |
|  | Bent u vandaag door een huisarts verwezen? | ⃝ Ja; ga naar vraag 7  ⃝ Nee; ga naar vraag 9 | |
|  | Hoe laat hebt u vandaag de huisarts gecontacteerd? | Tijdstip: ……:…… uur | |
|  | Hoe laat hebt u de huisarts vandaag bezocht, of kwam deze bij u thuis? | Tijdstip: ……:…… uur |  |
|  | Bent u vandaag met de ambulance naar de SEH gekomen? | ⃝ Ja; ga naar vraag 10  ⃝ Nee; ga naar vraag 11 | |
|  | Hoe laat was de ambulance vandaag bij u? | Tijdstip: ……:…… uur | |
|  | Tijdstip van binnenkomst SEH: | Tijdstip: ……:…… uur | |
|  | Tijdstip van vertrek SEH: | Tijdstip: ……:…… uur | |
|  | Welke diagnose is er uiteindelijk door de dokter op de SEH gesteld? | ………………………………………………………………………………. | |

| **UW ERVARING MET BETREKKING TOT HET TRAJECT VANDAAG** | |
| --- | --- |
|  | Hoe tevreden bent u over de duur van het traject tot nu toe? Dus vanaf het moment dat u vandaag contact zocht met een dokter tot en met de behandeling op de spoedeisende hulp?   \| Heel ontevreden \| 1 \| 2 \| 3 \| 4 \| 5 \| 6 \| 7 \| 8 \| 9 \| 10 \| Heel tevreden \| \| --- \| --- \| --- \| --- \| --- \| --- \| --- \| --- \| --- \| --- \| --- \| --- \| \|  \| ⃝ \| ⃝ \| ⃝ \| ⃝ \| ⃝ \| ⃝ \| ⃝ \| ⃝ \| ⃝ \| ⃝ \|  \| |
|  | Hoe tevreden bent u over de geboden kwaliteit tot nu toe? Dus vanaf het moment dat u vandaag contact zocht met een dokter tot en met de behandeling op de spoedeisende hulp?   \| Heel ontevreden \| 1 \| 2 \| 3 \| 4 \| 5 \| 6 \| 7 \| 8 \| 9 \| 10 \| Heel tevreden \| \| --- \| --- \| --- \| --- \| --- \| --- \| --- \| --- \| --- \| --- \| --- \| --- \| \|  \| ⃝ \| ⃝ \| ⃝ \| ⃝ \| ⃝ \| ⃝ \| ⃝ \| ⃝ \| ⃝ \| ⃝ \|  \| |
|  | Ziet u ruimte voor verbetering in het traject van vandaag? Dus vanaf het moment dat u vandaag contact zocht met een dokter tot en met de behandeling op de spoedeisende hulp?  ………………………………………………………………………………………………………………………………………………………………………………………  ………………………………………………………………………………………………………………………………………………………………………………………  ………………………………………………………………………………………………………………………………………………………………………………………  ………………………………………………………………………………………………………………………………………………………………………………………  ……………………………………………………………………………………………………………………………………………………………………………………… |

| VOORDAT U VANDAAG OP DE SPOEDEISENDE HULP KWAM | | | | |
| --- | --- | --- | --- | --- |
|  | Hoeveel dagen had u in totaal klachten voordat u vandaag kwam? | | | ⃝ Sinds vandaag klachten  ⃝ ………… dagen klachten  ⃝ langer dan 1 maand klachten |
|  | Hebt u sindsdien contact gehad met huisarts, ambulance, of een dokter in het ziekenhuis? | | | ⃝ Ja; ga naar vraag 19  ⃝ Nee; ga naar vraag 21 |
|  | Hoe vaak hebt u sindsdien contact gehad met huisarts, ambulance, of dokter in het ziekenhuis? | | | ……………… keer |
|  | Werd er al medicatie aan u voorgeschreven? | ⃝ Nee  ⃝ Ja, namelijk: …………………………………………… | | Datum: ……-……-…………  Tijdstip: ……:…… uur |
|  | Vindt u dat u op het goede moment naar de SEH gekomen / verwezen bent? | | ⃝ Ja  ⃝ Nee, ik had hier eerder moeten zijn  ⃝ Nee, ik had hier nu nog niet hoeven zijn | |

| **ALGEMEEN** | |
| --- | --- |
|  | Hebt u nog een vraag gemist die ons iets over uw ervaring tot nu toe had kunnen vertellen? Zo ja, welke vraag?  ………………………………………………………………………………………………………………………………………………………………………………………  ………………………………………………………………………………………………………………………………………………………………………………………  ………………………………………………………………………………………………………………………………………………………………………………………  ………………………………………………………………………………………………………………………………………………………………………………………  ……………………………………………………………………………………………………………………………………………………………………………………… |
