# Supplementary material for: From symptom onset to ED departure: understanding the acute care chain for patients with undifferentiated complaints: a prospective observational study
Source: Int J Emerg Med. 2024 Apr 15;17:55. doi: 10.1186/s12245-024-00629-x (PMC11020825; doi:10.1186/s12245-024-00629-x)
Supplement: Supplementary file 2 — Supplementary Material 2 [file 12245_2024_629_MOESM2_ESM.docx]

**Appendix B**

**Table 1.** Comparison of included with non-included patients

|  | Included patients (n = 286) | Non-included patients (n = 339) | P value |
| --- | --- | --- | --- |
| **Age in years**, mean (min-max) | 65 (IQR 52-75) | 66 (IQR 50-77) | .550 |
| **Sex**  Male  Female |  |  |  |
|  | 123 (43.0%) | 188 (55.6%) | .002 |
|  | 163 (57.0%) | 150 (44.4%) |  |
| **ED triage urgency** |  |  |  |
| Highly urgent | 73 (25.5%) | 109 (32.2%) | .052 |
| Urgent | 205 (71.7%) | 216 (63.7%) |  |
| Missing | 8 (2.8%) | 14 (4.1%) |  |

Values: n (%) or median (IQR)
